# Editorialets

**Published:** 1909-01

**Authors:** 


### Editorialets.

"It looks good to me." Keep it coming.—W. H. Seale, M. D.

---

I get the "Red Back" regularly and always delighted.—E. P. Cox, M. D.

---

Renew your subscriptions for 1908. The "Red Back" will keep up this gait, and if this number is not a whizzer, I don't want a cent.

---

Dr. E. Cross, a long time and foremost practitioner of San Antonio, has been induced to remove to Kingsville, Nueces county. We congratulate Nueces county. Our best wishes attend him.

---

Attention is called to the advertisement of the King Water Heater. It is the invention of Mr. Brooks Haynie, of Austin. I have one of them installed in my house, and am greatly pleased with it.

---

Miss Mary Hudson, "Daughter of the Texas State Medical Association" and only daughter of Dr. Taylor Hudson, ex-President of the Association, was married at Belton, Texas, November 23, 1908, to Dr. (Captain) Wilson Thompson Davidson, of the Medical Corps of the United States Army.

---

The Seventh Councilor District Medical Society met at Austin December 17th with a large attendance. Dr. M. A. Taylor, the Nestor of Texas medicine, 56 years a practitioner in Austin, was elected by acclamation President of the society. A banquet followed, Dr. Daniel acting as toastmaster.

---

An English-Chinese Lexicon of Medical Terms, prepared by Dr. Philip B. Cousland, has just been published in Shanghai. Though the author is an Englishman by birth, he has based his book largely upon the Medical Dictionary of Dr. George M. Gould, of Philadelphia, a high compliment to American scholarship. Dr. Cousland has recently published a translation of Professor Halliburton's edition of Kirke's Physiology.

VALEDICTORY.

NEW YORK, December 24, 1908.

In retiring from the agency field, Mr. D. A. O'Gorman desires to express his sincere appreciation of the cordial relations constantly maintained between your publication and his agency, and believes that the same good feeling will be continued to and by his successor, Dr. H. E. Lewis, already favoraby known to the medical publishers of these United States.

---

HUGO, OKLA., December 4, 1908.

*Dr. F. E. Daniel, Austin, Texas.*

DEAR DOCTOR: Enclosed please find check for eight dollars ($8), which pays up my past indebtedness for the "old reliable" and a little in advance.

When I get behind again please "rub it in" as you did in your last statement, but continue to send the "Red Back."

Yours truly,

.......................M. D.

---

MARLIN, TEXAS, December 2, 1908.

*The Texas Medical Journal, Austin, Texas.*

DEAR DOCTOR: Enclosed you will find my check for $—, as per enclosed statement and permission to continue the ad. a year.

There will be presented to the next Legislature a law similar to the Gant law of Arkansas prohibiting drumming and soliciting for doctors. A great many physicians in the State are interested and anxious to see it pass, for this "boosting" is a crying evil, not only in Marlin, Mineral Wells and other health resorts, but in all the large cities where strangers are liable to go for medical treatment. My reason for calling your attention to this is that you might find it acceptable as an item for your publication.

Wishing success to the JOURNAL, I remain,

Yours sincerely,

J. W. TORBETT, M. D.

---

"Uncle Dick, how many toddies does the colonel drink every day?"

"Well, yo' see, boss, I eats de sugar de kunnel leaves in de glass, an' 'long erbout de middle of de evenin' I gets fuddled an' loses count."—*Lippincott's.*

